# Synthesis, Structure and Antibacterial Activity of Potent DNA Gyrase Inhibitors: N′-Benzoyl-3-(4-Bromophenyl)-1*H*-Pyrazole-5-Carbohydrazide Derivatives

**DOI:** 10.1371/journal.pone.0069751

**Published:** 2013-07-29

**Authors:** Juan Sun, Peng-Cheng Lv, Yong Yin, Rong-Ju Yuan, Jian Ma, Hai-Liang Zhu

**Affiliations:** 1 State Key Laboratory of Pharmaceutical Biotechnology, Nanjing University, Nanjing, China; 2 School of Life Sciences, Shandong University of Technology, Zibo, China; University of Edinburgh, United Kingdom

## Abstract

A total of 19 novel (**3a–3s**) N′-benzoyl-3-(4-bromophenyl)-1*H*-pyrazole-5-carbohydrazide analogs were designed, synthesized, and evaluated for biological activities as potential DNA gyrase inhibitors. The results showed that compound **3k** can strongly inhibit *Staphylococcus aureus* DNA gyrase and *Bacillus subtilis* DNA gyrase (with IC_50_ of 0.15 µg/mL and 0.25 µg/mL, respectively). Structure-activity relationships were also discussed base on the biological and docking simulation results.

## Introduction

Over the past decade, bacterial DNA gyrase has drawn much attention as a selected target for finding potent antibacterial agents [1]–[6]. DNA gyrase is mainly inhibited by quinolones and coumarins, some of which are widely used for the treatment of bacterial infectious diseases (e.g., ciprofloxacin) [7]–[11]. However, because of side effects, no pharmaceutically useful drug has so far been derived from the coumarins. Recently, multidrug-resistant Gram-positive bacteria, such as methicillin-resistant *Staphylococcus aureus* (MRSA), penicillin-resistant *Streptococcus pneumonia* (PRSP), and vancomycin-resistant enterococci (VRE), have become a serious medical problem [12]. Since most of these multidrug-resistant bacteria are also quinolone-resistant ones, it is important to find a new class of DNA gyrase inhibitors to solve this problem.

**Figure 1:**
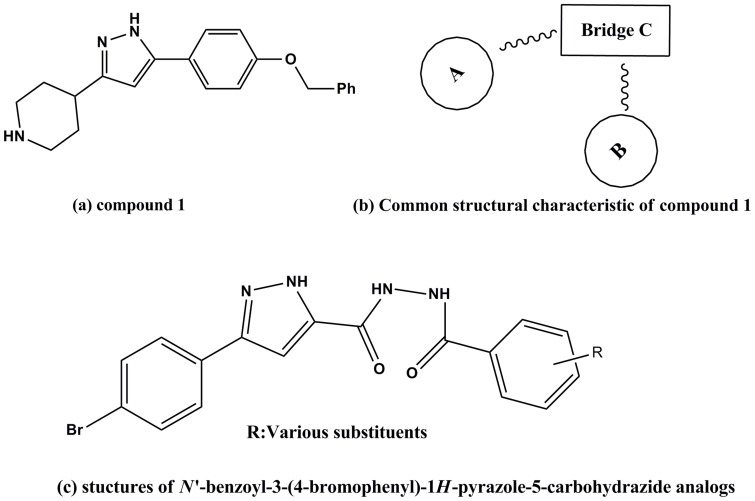
Structure of compound 1 and N′-benzoyl-3-(4-bromophenyl)-1*H*-pyrazole-5-carbohydrazide analogs and common structural characteristic of compound 1.

Many pyrazole derivatives are well acknowledged to possess a wide range of antibacterial bioactivities [13]–[18]. Tanitame et al. [12] have found compound 1 (Figure 1a) as potent and selective inhibitor of DNA gyrase. For the sake of simplicity, here the molecule is subdivided into three main parts (ring A, ring B, and the bridge ring C; Figure 1b). Various structural modifications to this compound have been reported. The structural modifications to the bridge ring C are topics of most concern. Most of this series of derivatives retain both cytotoxic and DNA gyrase inhibitory activity, and a number of derivatives were found to be slightly more potent than compound **1** itself [19]–[21]. Ring B is also received greater attentions by medicinal chemists. Substituents on Ring B of the compound **1** can interact with the amino acid residues of DNA gyrase and thus inhibit its activity accordingly. However, to the best of our knowledge, few reports have been dedicated to the synthesis and DNA gyrase inhibitory activity of pyrazole derivatives maintaining the bridge ring C unchanged.

**Figure 2:**
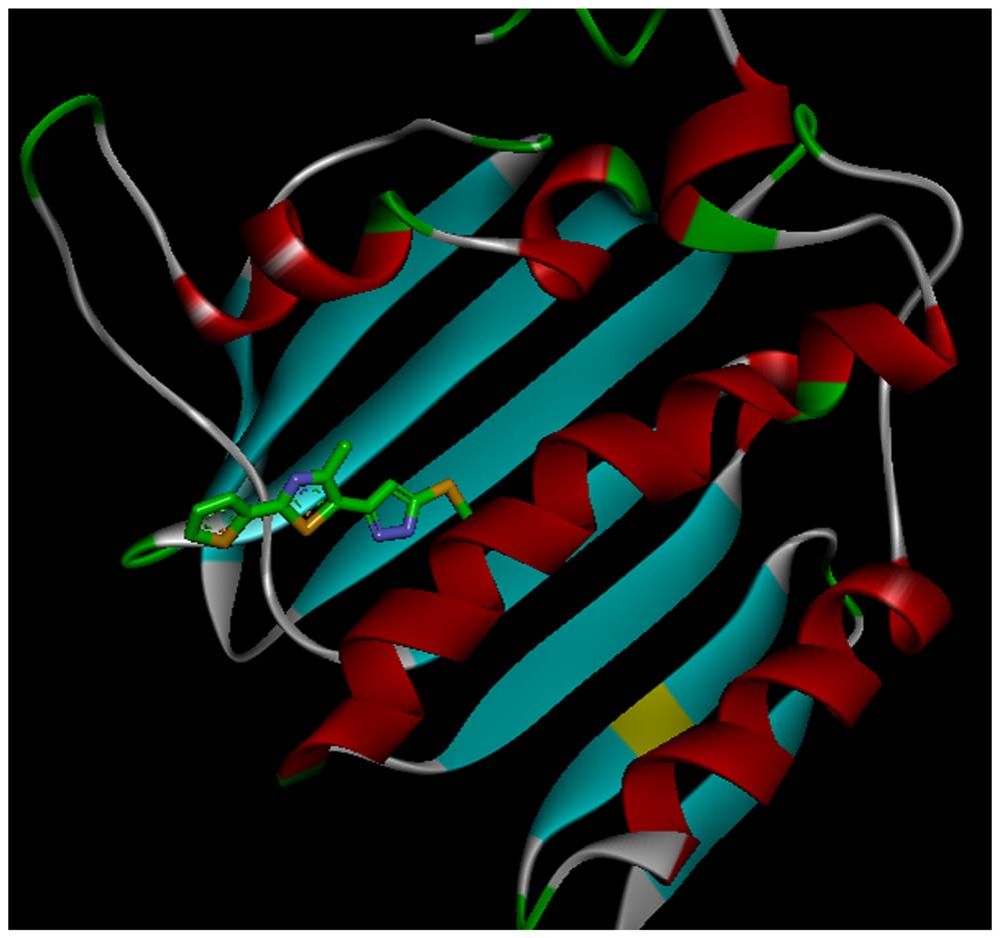
Crystal structure of *Staphylococcus aureus* DNA gyrase co-complexed with inhibitor.

In view of the above mentioned findings, herein, we report in the present work the design and synthesis of a series of N′-benzoyl-3-(4-bromophenyl)-1*H*-pyrazole-5-carbohydrazide derivatives (Table 1) to extend the research to achieve new potential antibacterial DNA gyrase inhibitors with the aim to find new DNA gyrase inhibitors as antibacterial agents. Docking simulations were performed using the X-ray crystallographic structure of the DNA gyrase of *Staphylococcus aureus*, which was shown in Figure 2, in complex with the most potent inhibitor to explore the binding model of the compound at the enzyme active site.

**Table 1:** Chemical structures of 3a–3s.

compound	R^1^	R^2^	R^3^
**3a**	H	H	F
**3b**	H	H	Cl
**3c**	H	H	Br
**3d**	H	H	CH_3_
**3e**	H	H	CH_3_O
**3f**	H	H	NO_2_
**3g**	H	F	H
**3h**	H	Cl	H
**3i**	H	Br	H
**3j**	H	CH_3_	H
**3k**	H	CH_3_O	H
**3l**	H	NO_2_	H
**3m**	F	H	H
**3n**	Cl	H	H
**3o**	Br	H	H
**3p**	CH_3_	H	H
**3q**	CH_3_O	H	H
**3r**	NO_2_	H	H
**3s**	H	H	H

## Results and Discussion

### Chemistry

**Figure 3:**
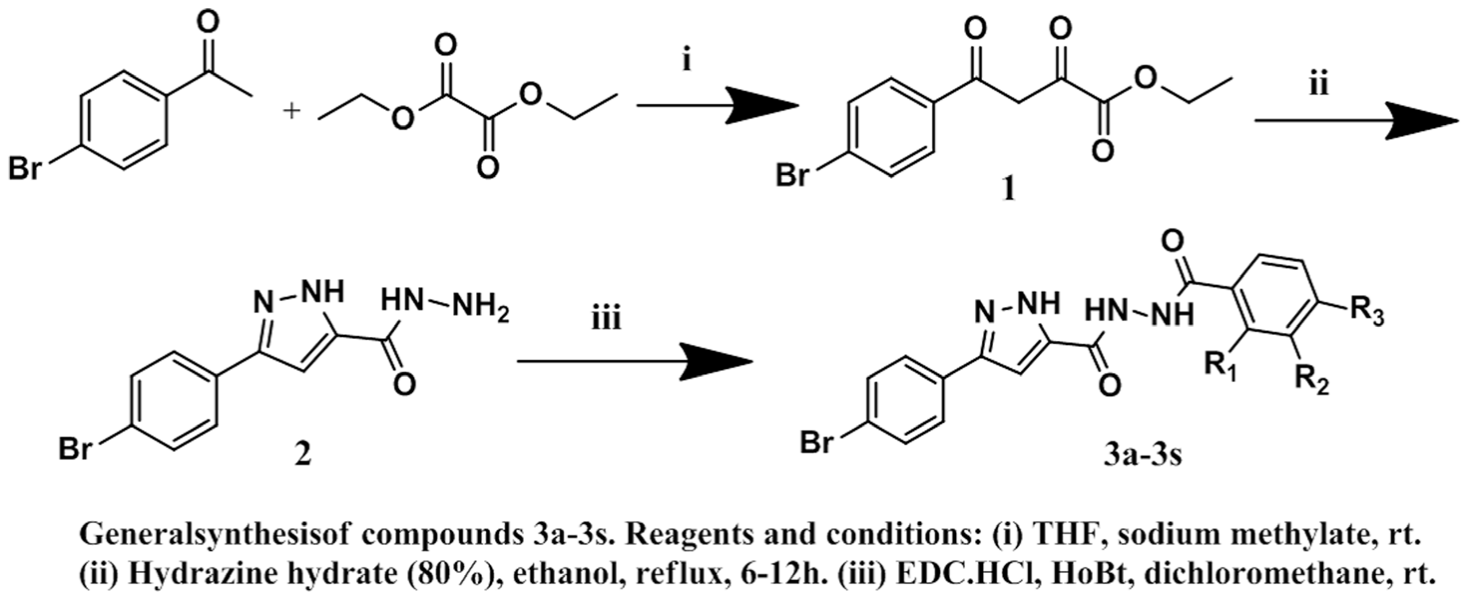
General synthesis of compounds 3a–3s.

The synthetic route to target compounds (**3a**–**3s**) is shown in Figure 3. The synthesis of ester **1** was carried out using a literature method [22], by reaction of commercially available bromoacetophenone and dimethyl oxalate in the presence of sodiumhydride with excellent yield. Treatment of **1** with anhydrous hydrazine [23] yielded pyrazolehydrazide **2** which can be condensed with various substituted benzoic acids under standard conditions [24] provided the desired DNA gyrase inhibitors (**3a–3s**) with good yield.

All of the synthetic compounds gave satisfactory analytical and spectroscopic data, which were full accordance with their depicted structures.

### In vitro antibacterial assay

The activities of synthesized compounds were tested against *Bacillus subtilis* ATCC 6633, *Escherichia coli* ATCC 35218, *Pseudomonas aeruginosa* ATCC 27853 and *Staphylococcus aureus* ATCC 6538 which may be causal agents of some serious infections in humans using MH medium (Mueller-Hinton medium: casein hydrolysate 17.5 g, soluble starch 1.5 g, beef extract 1000 mL). The MICs of the compounds against four bacteria are presented in Table 2. Also included are the activities of reference compounds kanamycin. The results revealed that some of the synthesized compounds exhibited significant antibacterial activity, especially against *B. subtilis* ATCC 6633 and *S. aureus* ATCC 6538.

**Table 2:** Antimicrobial activity of the synthesized compounds.

Compounds	Minimum inhibitory concentrations (µg/mL)			
	*B. subtilis*	*S. aureus*	*P.aeruginosa*	*E. coli*
**3a**	12.52	15.00	50	>50
**3b**	17.12	15.00	50	>50
**3c**	10.21	13.79	50	>50
**3d**	12.58	3.66	12.50	50
**3e**	10.12	6.68	12.50	50
**3f**	25.00	18.03	>50	>50
**3g**	25.00	13.00	50	>50
**3h**	6.25	12.56	50	>50
**3i**	12.58	12.11	50	>50
**3j**	5.32	1.12	12.50	50
**3k**	3.12	0.78	12.50	50
**3l**	10.78	10.78	50	>50
**3m**	25.0	22.10	>50	>50
**3n**	12.24	16.97	>50	>50
**3o**	23.12	20.99	>50	>50
**3p**	12.15	8.08	12.50	50
**3q**	20.58	18.71	>50	>50
**3r**	50	25.00	>50	>50
**3s**	10.56	10.56	12.50	12.50
**Penicillin**	1.56	1.56	6.25	6.25

The compounds **3j** and **3d** showed antibacterial activities against *B. subtilis* with the MIC of 1.12, 3.66 µg/mL, respectively, comparable to that of positive control penicillin. Compound **3k** with MIC value of 0.78 µg/mL exhibited promising antibacterial activities against *B. subtilis* which were even better than that of the commercial penicillin. The compounds **3d**, **3e**, **3j**, **3k**, **3p** and **3s** showed moderate antibacterial activities against *P. aeruginosa* with MIC of 12.50 µg/mL. Besides, compound **3s** also showed moderate antibacterial activities against *E. coli* with MIC of 12.50 µg/mL.

From the structure-activity relationships presented in Table 2, it can be concluded that some N′-benzoyl-3-(4-bromophenyl)-1*H*-pyrazole-5-carbohydrazide derivatives showed good activity against Gram positive strains (*B. subtilis* ATCC 6633 and *S. aureus* ATCC 65385), but most of the derivatives displayed poor activity against Gram negative strain (*P. aeruginosa* ATCC 27853 and *E. coli* ATCC 35218).

Among all the synthetic compounds, we found a law between the compounds and antibacterial activity of *S. aureus*. In general, compounds with electronic-donating substituents (methyl or methoxy) on the benzene ring showed more potent inhibitory activities than compounds only contained electronic-withdrawing substituents (halogen) on the benzene ring. Exceptionally compound **3q** with methoxy on the benzene ring exhibited lower antibacterial activity compared with other compounds. The position of the same substituents on benzene ring also influenced the activities. For example, the order of the activities is that substituent at the *meta* position>substituent at the *para* position>substituent at the *ortho* position.

### DNA gyrase inhibitory assay

To elucidate the mechanism by which the pyrazole derivatives induce antibacterial activity, the inhibitory activities of selected compounds were examined against DNA gyrase isolated from *B. subtilis* and *S. aureus*. As shown in Table 3, compound **3k** with potent antibacterial activities strongly inhibited *S. aureus* DNA gyrase and *B. subtilis* DNA gyrase (with IC_50_ of 0.15 µg/mL against *S. aureus* DNA gyrase, 0.25 µg/mL against *B. subtilis* DNA gyrase). There was a good correlation between the MICs and the IC_50_ (Tables 2 and 3), indicating that inhibition of the DNA gyrase by the pyrazole derivatives caused inhibition of bacterial cell growth. Bacterial topoisomerase inhibitors sometimes have poor selectivity against human topoisomerase, for example, the compound **3s** showed the same activities against *S. aureus* and *B. subtilis* with the MIC of 10.56 µg/mL, but it showed different inhibition against the *S. aureus* DNA gyrase and *B. subtilis* DNA gyrase (IC_50_ = 3.25 µg/mL, 1.00 µg/mL respectively).

**Table 3:** Inhibitory effects of the selected title compounds against DNA gyrase.

Compounds	IC_50_(µg/mL)	
	*S.aureus* DNA gyrase	*B.subtilis* DNA gyrase
**3d**	1.50	2.60
**3e**	3.40	12.25
**3j**	0.13	3.25
**3k**	0.15	0.25
**3p**	5.21	0.50
**3s**	3.25	1.00
**Novobiocin**	0.25	0.5

In addition, we did activity correlation analysis between antibacterial and anti DNA gyrase. They were positively correlated with R^2^ = 0.8097.

### Molecular docking study of synthetic compounds

**Figure 4:**
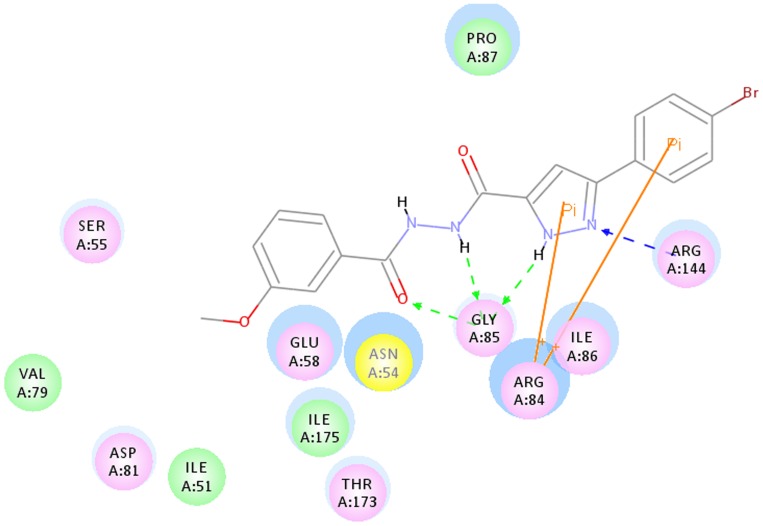
2D Ligand interaction diagram of compound 3k with DNA gyrase.

**Figure 5:**
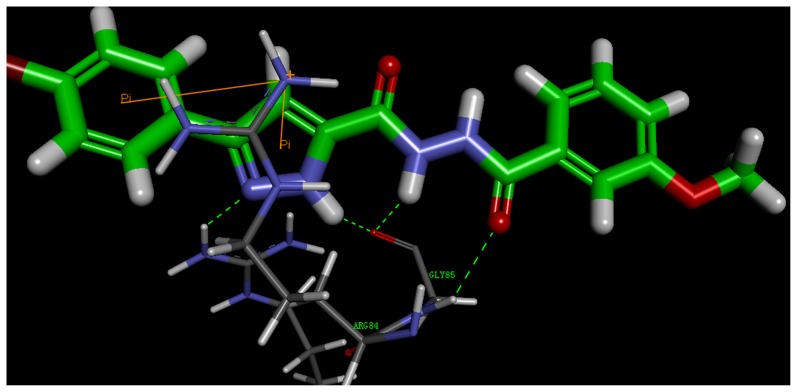
3D model of the interaction between compound 3k and DNA gyrase binding site.

To help understand the SARs observed at the DNA gyrase and guide further SAR studies, we proceeded to examine the interaction of compound **3k** with DNA gyrase (PDB code: 3G75). All docking runs were applied the CDOCKER protocol in Discovery Studio 3.1 (Discovery Studio 3.1, Accelrys, Inc., San Diego, CA). The obtained results were presented in the group of pictures. Figure 4 and 5 showed the binding mode of compound **3k** interacting with DNA gyrase and the docking results revealed that two amino acids ARG 144, GLY 85 and ARG 84 located in the binding pocket played vital roles in the conformation with compound **3k**, which were stabilized by two *π*-cation bonds and four hydrogen bonds that showed in 2D diagram. One *π*-cation bond with 5.50 Å was formed between amino acid AGR84 and benzene ring of compound **3k**; one *π*-cation bond, of which its length was 3.97 Å, was formed by the pyrazole ring and ARG84. The nitrogen atom on pyrazole ring provided one hydrogen bond with ARG144 (N…H-N: 2.20 Å, 143.72°). The other hydrogen bonds were formed between GLY 85 and carbonyl oxygen (N…H-O: 2.24 Å, 120.69°), amino hydrogen (O-H: 2.44 Å, 119.96°) and hydrogen atom on pyrazole ring (O-H: 2.12 Å, 127.68°).

**Figure 6:**
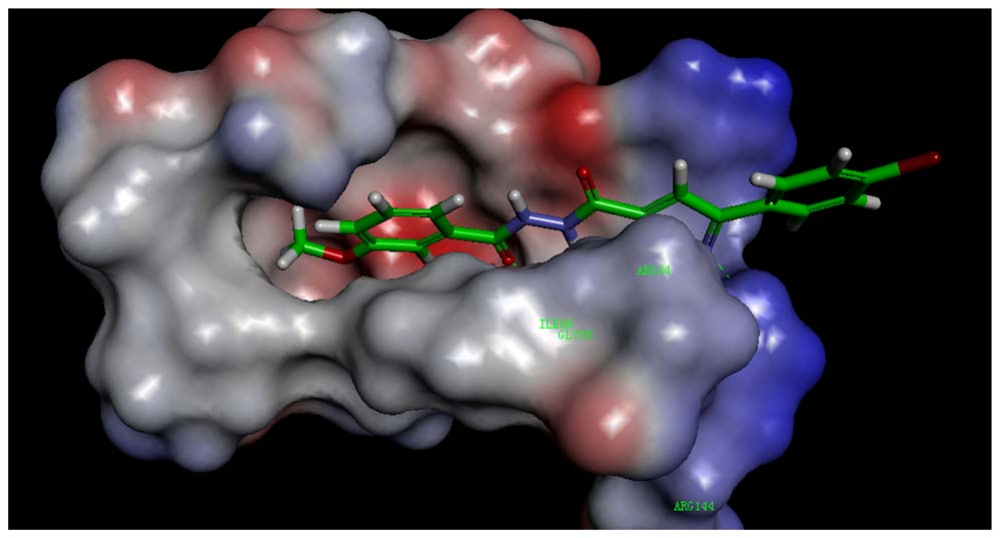
The receptor surface model with compound 3k.

In addition, the enzyme surface model was showed in Figure 6, which revealed that the molecule was well embedded in the active pocket. Docking result along with the antibacterial activity date, suggested that compound **3k** was a potential inhibitor of DNA gyrase. The docking calculations of the other compounds were also depicted in Table 4.

**Table 4:** The docking calculation of the synthesized compounds (3a–3s).

Compound	-CDOCKER_ENERGY
**3a**	17.3167
**3b**	17.4755
**3c**	17.7234
**3d**	20.0936
**3e**	19.7220
**3f**	14.3571
**3g**	17.7452
**3h**	18.1927
**3i**	18.4996
**3j**	20.6595
**3k**	22.7154
**3l**	19.3675
**3m**	10.8604
**3n**	16.1455
**3o**	11.6705
**3p**	17.5309
**3q**	14.0062
**3r**	6.4841
**3s**	19.4014

The difference in the target compounds is just their substituent, and therefore, their binding modes are substantially identical. Since they are substantially the same, the difference exists in the substituent. Some substituents can form better interactions, so that the combination is enhanced. Some affect the activity by influencing peripheral electronic arrangement. Overall, these are reflected in the binding energy of this parameter. In support of this, we did an activity correlation analysis between docking calculations and anti DNA gyrase activity. They were positively correlated with R^2^ = 0.8045.

### Crystal structures of compounds 3n

**Figure 7:**
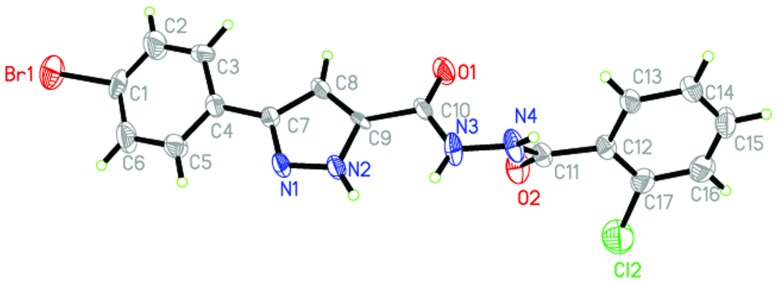
Molecular structure of compound 3n with atomic numbering scheme.

**Figure 8:**
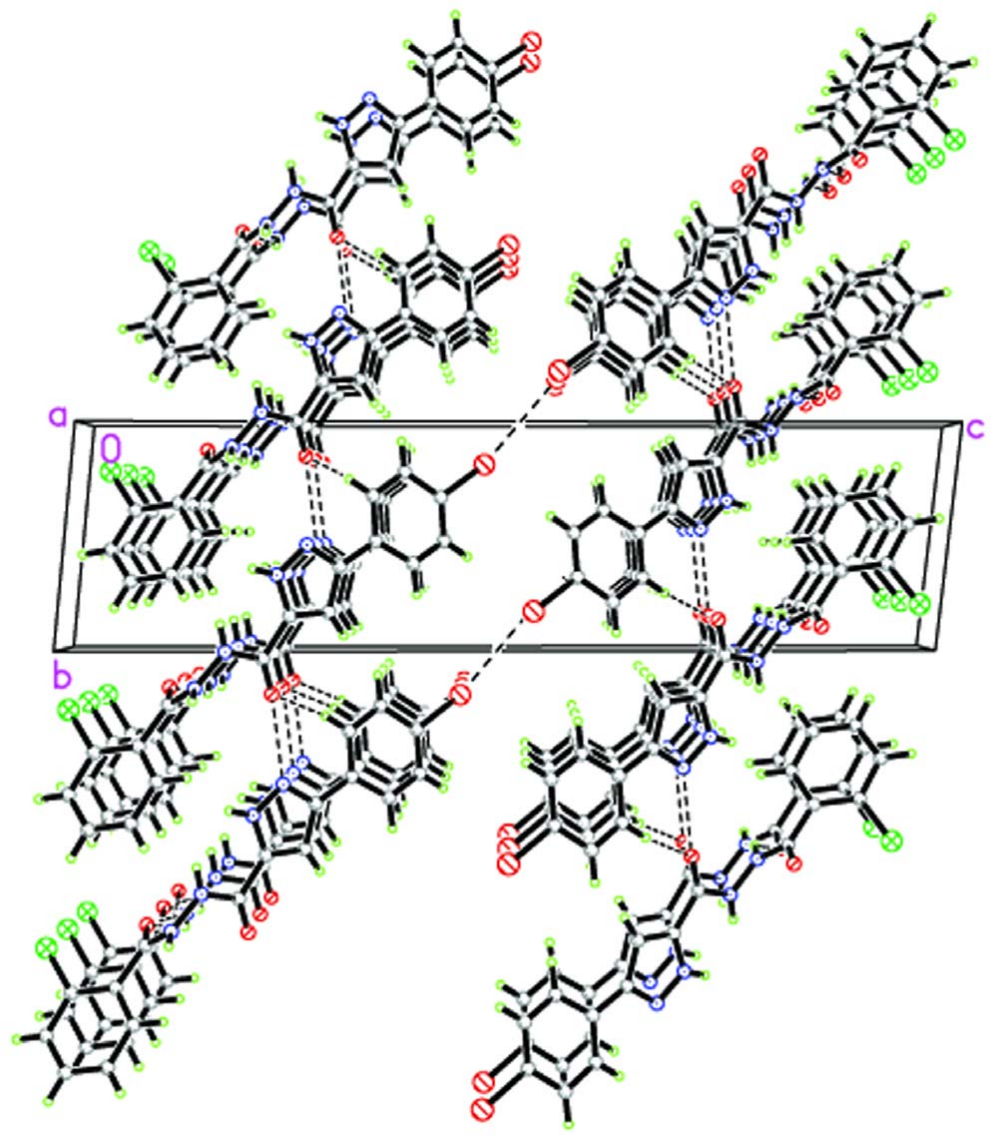
Crystal packing of the compound 3n.

Crystals of compound **3n** were obtained from methanol solution. Figure 7 shows a perspective view of the monomeric unit with the atomic numbering scheme, and Figure 8 depicts the intramolecular and intermolecular hydrogen bonds. Crystallographic data, details of data collection and structure refinement parameters are listed in Table 5. The hydrogen bond lengths and bond angles are given in Table 6.

**Table 5:** Crystallographic data, details of data collection and structure refinement parameters.

compound	3n
Empirical formula	C_17_H_12_BrClN_4_O_2_
Formula weight	419.66
Crystal system	Triclinic
Space group	P-1
*a* (Å)	4.6083(12)
*b* (Å)	7.0246(18)
*c* (Å)	26.804(7)
*α* (°)	95.804(8)
*β* (°)	92.071(8)
*γ* (°)	95.433(8)
*V* (Å)	858.4(4)
*Z*	2
D calc/g cm^−3^	1.624
θ range (o)	2.3–25.5
*F*(000)	420
Reflections collected/unique	8021, 3148
Data/restraints/parameters	1686/0/226
Absorption coefficient (mm^−1^)	2.569
*R*_1_/*_W_R*_2_ [*I*>2σ (*I*)]	0.0994/0.2380
*R*_1_/*_W_R*_2_ (all date)	0.1762/0.2662
GOOF	1.106

**Table 6:** Hydrogen Bond Lengths (Å) and Bond Angles (°) of compound 3n.

D–H…A	d(D–H)	d(H…A)	d(D…A)	□∠DHA
N(3)…H(3)…N(2)	0.86	2.36	2.720(12)	105
N(4)…H(4)…O(2)	0.86	2.02	2.732(11)	140
C(5)…H(5)…O(1)	0.93	2.54	3.409(15)	156
C(5)…H(5)…N(1)	0.93	2.60	2.922(15)	101

Single crystal of **3n** (0.32 mm×0.27 mm×0.25 mm) was mounted on a *D*-8 venture diffractometer equipped with graphite-monochromated MoKa (*λ* = 0.71073 Å) radiation. For **3n**, a total of 8021 reflections were collected, of which 3148 were unique with R_int_ = 0.073 and 1686 observed reflections with I>2*σ* (I) were used in the succeeding structure calculations. The final cycle of refinement of full matrix least-squares was converged to R = 0.0994 and *w*R = 0.2662. The highest and lowest residual peaks in the final difference Fourier map are 0.66 and −0.41 e/Å^3^, respectively.

In the crystal structure of compound **3n**, there are two benzene rings in the molecule. C(1), C(2), C(3), C(4), C(5) and C(6) form the first plane with the mean deviation of 0.0145 Å, defined as plane I; Similarly, C(12), C(13), C(14), C(15), C(16) and C(17) forms the second plane with the mean deviation of 0.0021 Å, defined as plane II. The dihedral angle between plane I and plane II is 55.5°. Besides, there is one pyrazole ring in the molecule, C(7), C(8), C(9), N(2) and N(1) form the third plane with the mean deviation of 0.0051 Å, defined as plane III. The dihedral angle between plane I and plane III is 17.0°, the dihedral angle between plane II and plane III is 68.2°.

**Figure 9:**
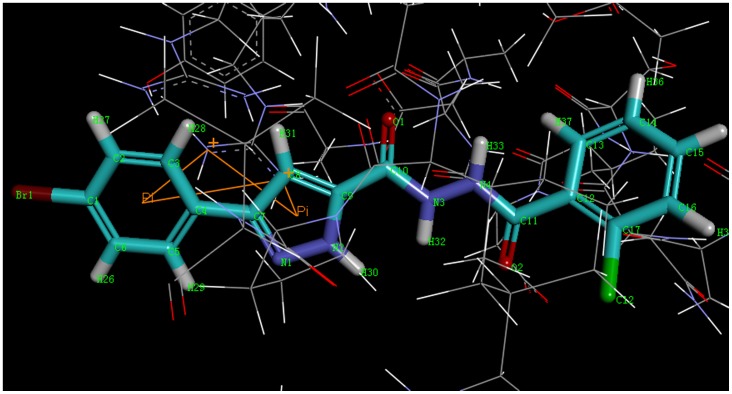
3D model of compound 3n.

In addition, a connection in terms of structural aspects between theoretical results from docking calculations and X-ray data for compound **3n** is also explored. Figure 9 showed the binding mode of compound **3n** interacting with DNA gyrase. The main bond lengths and bond angles of docking calculations and X-ray data are given in Table 7 and Table 8. There fitting degree are 0.9597 and 0.8565, respectively.

**Table 7:** Bond lengths from docking calculations and X-ray data for compound 3n.

Bond	Bond lengths (Å)	
	Docking data	X-ray data
C2-Br1	1.878	1.902
C4-C7	1.488	1.472
C7-C8	1.403	1.376
C8-C9	1.401	1.389
C9-N2	1.341	1.310
N1-N2	1.355	1.314
N1-C7	1.365	1.355
C9-C10	1.384	1.483
C10-O1	1.225	1.174
C10-N3	1.343	1.357
N3-N4	1.236	1.372
N4-C11	1.347	1.329
C11-O2	1.225	1.218
C11-C12	1.474	1.484
C17-Cl2	1.749	1.708

**Table 8:** Bond Angles from docking calculations and X-ray data for compound 3n.

Angle	Bond Angles (°)	
	Docking data	X-ray data
C2-C1-Br1	120.48	121.6
C3-C4-C7	121.78	121.3
C5-C4-C7	119.97	120.6
C9-C10-O1	120.59	121.5
C9-C10-N3	115.53	113.5
C10-N3-N4	121.63	119.4
O1-C10-N3	123.88	124.9
O2-C11-C12	124.01	123.2
C11-C12-C13	119.60	119.4
C11-C12-C17	122.43	122.5
C16-C17-Cl2	115.88	119.1

### Conclusion

Using the structure-based drug design concept, a series of new N′-benzoyl-3-(4-bromophenyl)-1*H*-pyrazole-5-carbohydrazidederivatives (**3a**–**3s**) were designed and synthesized based on the molecular docking information. These compounds were evaluated and assayed for their antibacterial (*B. subtilis* ATCC 6633, *E. coli* ATCC 35218, *P. aeruginosa* ATCC 27853 and *S. aureus* ATCC 6538) activities by MTT method. The results show that compound **3k** possess potent antibacterial activity and can strongly inhibit *S. aureus* DNA gyrase and *B. subtilis* DNA gyrase, with IC_50_ of 0.15 µg/ml against *S. aureus* DNA gyrase, 0.25 µg/ml against *B. subtilis* DNA gyrase. The data of antibacterial activity and molecular docking are positively correlated with R value of 0.8045. The antibacterial activity and anti DNA gyrase inhibitory activity are also positively correlated as well, which has the R value of 0.8097.

## Experimental

### Chemistry

All chemicals and reagents used in the current study were of analytical grade. The reactions were monitored by thin layer chromatography (TLC) on Merck pre-coated silica GF254 plates. Melting points (uncorrected) were determined on a XT4MP apparatus (Taike Corp., Beijing, China). ESI mass spectra were obtained on a Mariner System 5304 mass spectrometer, and ^1^H NMR spectra were collected on a Bruker DPX300 spectrometer at room temperature with TMS and solvent signals allotted as internal standards. Chemical shifts are reported in ppm (*δ*). Elemental analyses were performed on a CHN-O-Rapid instrument, and were within±0.4% of the theoretical values.

### Synthesis of ethyl 4-(4-bromophenyl)-2,4-dioxobutanoate (1)

To a suspension of sodium methylate (5.4 g, 100 mmol) in methanol (5 mL) at 0°C was added tetrahydrofuran (50 mL) slowly. To this cold mixture was added a solution of 1-(4-bromophenyl) ethanone (9.9 g, 50 mmol) and dimethyl oxalate (8.76 g, 60 mmol) in tetrahydrofuran (150 mL) dropwise. The mixture was allowed to warm to room temperature, stirred overnight and filtered. The residue was purified through washing several times to yield compound **1** as a yellow solid.

### Synthesis of 3-(4-bromophenyl)-1*H*-pyrazole-5-carbohydrazide (2)

Hydrazine hydrate (5.32 mL, 200 mmol) was added to a suspension of **1** (5.96 g, 20 mmol) in EtOH (250 mL) and the mixture was refluxed overnight. The precipitated white solid was filtered, washed with EtOH and dried under vacuum to yield compound **2** as a white solid.

### General procedure for the preparation of target compounds 3a–3s

A stirred solution of compound **2** (0.1 mol) in CH_2_Cl_2_ (50 mL) was treated with the appropriate substituted benzoic acid, EDC.HCl (0.15 mol), HOBt (0.05 mol) and refluxed overnight. Then purification with recrystallisation afforded the corresponding compound.

### Bioassay conditions

#### In vitro antibacterial activity

The antibacterial activity of the synthesized compounds was tested against *B. subtilis*, *E. coli*, *P. aeruginosa* and *S. aureus* using MH medium (Mueller-Hinton medium: casein hydrolysate 17.5 g, soluble starch 1.5 g, beef extract 1000 mL). The MICs (minimum inhibitory concentrations) of the test compounds were determined by a colorimetric method using the dye MTT (3-(4,5-dimethylth-iazol-2-yl)-2,5-diphenyl tetrazoliumbromide). A stock solution of the synthesized compound (100 µg/mL) in DMSO was prepared and graded quantities of the test compounds were incorporated in specified quantity of sterilized liquid MH medium. A specified quantity of the medium containing the compound was poured into microtitration plates. Suspension of the microorganism was prepared to contain approximately 10^5^ cfu/mL and applied to microtitration plates with serially diluted compounds in DMSO to be tested and incubated at 37°C for 24 h. After the MICs were visually determined on each of the microtitration plates, 50 µL of PBS (phosphate buffered saline 0.01 mol/L, pH 7.4, Na_2_HPO_4_·12H_2_O 2.9 g, KH_2_PO_4_ 0.2 g, NaCl 8.0 g, KCl 0.2 g, distilled water 1000 mL) containing 2 mg of MTT/mL was added to each well. Incubation was continued at room temperature for 4–5 h. The content of each well was removed, and 100 µL of isopropanol containing 5% 1 mol/L HCl was added to extract the dye. After 12 h of incubation at room temperature, the optical density (OD) was measured with a microplate reader at 550 nm.

### Enzyme inhibition

#### *S. aureus* DNA gyrase supercoiling

The *S. aureus* DNA gyrase were purified by the F. Blanche [25] from a crude extract of *S. aureus* and cultivated with medium B, which was composed of 10 g of polypeptone, 2 g of yeast extract, 8 g of Na_2_HPO_4_, 2 g of KH_2_PO_4_, 1.2 g of (NH_4_)_2_SO_4_, 0.2 g of MgSO_4_, 4 g of glucose perliter of distilled water. Supercoiling and decatenation were performed according to F. B lanche [25].

#### *B. subtilis* DNA gyrase supercoiling

The *B. subtilis* DNA gyrase were purified by the methods of E. Orr. [26]: Cells were suspended in an equal volume of 25 mM HEPES-KOH (pH 8.0)-100 mM KCl and stored frozen at −70°C. The frozen cell suspension was thawed and diluted with an equal volume of 25 mM HEPES-KOH (pH 8.0)-0.4 M sucrose-20 mM magnesium acetate-1 mM dithiothreitol-5 mM PMSF. All operations were performed at 0–4°C. Lysozyme was added to a final concentration, and the mixture was incubated for 2.5 h. One-third volume of 2 M KCl-1.5% Brij was added, and the incubation was continued for 15 min. The lysate was then centrifuged for 90 min intirotor. The supernatant was adjusted to a KCl concentration of 0.2 M by dilution with 25 mM HEPES-KOH (pH 8.0)-1 mM dithiothreitol-1mMEDTA-0.5 mM pmsf-10% ethylene glycol and applied to a column. The column was washed with starting buffer and eluted successively with buffer containing 20 mM ATP-25 mM magnesium acetate-0.2 M KCI, buffer (2 M KCI), and 5 M urea in buffer (0.2 M KCl).Protein-containing fractions were dialyzed against buffer (0.05 MKCI). Supercoiling and decatenation were carried out by the F. Blanche [25] method.

### Experimental protocol of docking study

Automated docking studies were carried out using Discovery Studio (version 3.1) as implemented through the graphical user interface DS-CDocker protocol [27].

The three-dimensional structures of the aforementioned compounds were constructed using Chem. 3D ultra 11.0 software [Chemical Structure Drawing Standard; Cambridge Soft corporation, USA (2009)], then they were energetically minimized by using MOPAC with 100 iterations and minimum RMS gradient of 0.10. The Gasteiger-Hückel charges of ligands were assigned. The crystal structures of DNA gyrase (PDB code: 3G75) complex were retrieved from the RCSB Protein Data Bank (http://www.rcsb.org/pdb/home/home.do). For enzyme preparation, the hydrogen atoms were added with the pH of the protein in the range of 6.5–8.5. CDOCKER is an implementation of a CHAR Mm based molecular docking tool using a rigid receptor. It includes the following steps:

1. A series of ligand conformations are generated using high temperature molecular dynamics with different random seeds.
2. Random orientations of the conformations are generated by translating the center of the ligand to a specified position within the receptor active site, and making a series of random rotations. A softened energy is calculated and the orientation is kept when it is less than a specified limit. This process repeats until either the desired number of low-energy orientations is obtained, or the test times of bad orientations reached the maximum number.
3. Each orientation is subjected to simulated annealing molecular dynamics. The temperature is heated up to a high temperature then cooled to the target temperature. A final energy minimization of the ligand in the rigid receptor using non-softened potential is performed.
4. For each of the final pose, the CHARMm energy (interaction energy plus ligand strain) and the interaction energy alone are figured out. The poses are sorted according to CHARMm energy and the top scoring (most negative, thus favorable to binding) poses are retained. The whole DNA gyrase domain defined as a receptor and the site sphere was selected based on the ligand binding location of B-482, then the B-482 removed and the ligands prepared by us was placed during the molecular docking procedure. CHARMm was selected as the force field. The molecular docking was performed with a simulated annealing method. The heating steps were 2000 with 700 of heating target temperature. The cooling steps were 5000 with 300 cooling target temperature. Ten molecular docking poses saved for each ligand were ranked according to their dock score function. The pose with the highest -CDOCKER energy was chosen as the most suitable pose.

### X-ray crystallography

Single crystal *X*-ray diffraction data was collected on a Bruker *D*-8 venture diffractometer at room temperature (293 K). The *X*-ray generator was operated at 50 KV and 35 mA using Mo Kα radiation (*λ* = 0.71073 Å). The data was collected using SMART software package. The data were reduced by SAINT-PLUS, an empirical absorption correction was applied using the package SADABS and XPREP were used to determine the space group. The crystal structure was solved by direct methods using SIR92 and refined by full-matrix least-squares method using SHELXL97 [28], [29]. All non-hydrogen atoms were refined anisotropic ally and hydrogen atoms have been refined in the riding mode on their carrier atoms wherever applicable.

## Supporting Information

File S1 **Experimental protocols, NMR data (^1^H and ^13^C), Mass spectrometry data (MS and HRMS) and Melting points data of compounds.** The connection between theoretical results from docking calculations and X-ray data for compound 3n (graph 1 and graph 2). Correlations among antibacterial, anti DNA gyrase and CDOCKER-ENERGY (graph 3 and graph 4).
(DOC)
