# Supplementary material for: Synthesis, Structure and Antibacterial Activity of Potent DNA Gyrase Inhibitors: N′-Benzoyl-3-(4-Bromophenyl)-1H-Pyrazole-5-Carbohydrazide Derivatives
Source: PLoS One. 2013 Jul 29;8(7):e69751. doi: 10.1371/journal.pone.0069751 (PMC3726784; doi:10.1371/journal.pone.0069751)
Supplement: File S1 — Experimental protocols, NMR data (1H and 13C), Mass spectrometry data (MS and HRMS) and Melting points data of compounds. The connection between theoretical results from docking calculations and X-ray data for compound 3n (graph 1 and graph 2). Correlations among antibacterial, anti DNA gyrase and CDOCKER-ENERGY (graph 3 and graph 4). (DOC) [file pone.0069751.s001.doc]

**File S1**

**Synthesis of 3-(4-bromophenyl)-1*H*-pyrazole-5-carbohydrazide (2)**

Hydrazine hydrate (5.32 mL, 200 mmol) was added to a suspension of **1** (5.96 g, 20mmol) in EtOH (250 mL) and the mixture was reﬂuxed overnight. The precipitated white solid was ﬁltered, washed with EtOH and dried under vacuum to yield compound **2** as a white solid.

White powder, mp: 158-160^o^C. ^1^H NMR (300 MHz, DMSO): 3.36-3.38 (m, 2H), 7.80-7.83 (m, 2H), 8.04-8.07 (m, 1H), 8.26-8.29 (d, *J* = 9.0 Hz, 1H), 8.89 (s, 2H), 13.37 (s, 1H). MS (ESI): 281 (C_10_H_10_BrN_4_O, [M+H]^+^). Anal.Calcd for C_10_H_9_BrN_4_O: C, 42.73; H, 3.23; N, 19.93%. Found: C, 42.81; H, 3.16; N, 19.82%.

**General procedure for the preparation of target compounds 3a-3s.**

A stirred solution of compound **2** (0.1 mol) in CH_2_Cl_2_ (50 mL) was treated with the appropriate substituted benzoic acid, EDC.HCl (0.15 mol), HOBt (0.05 mol) and reﬂuxed overnight. Then puriﬁcation with recrystallisation afforded the corresponding compound.

**3-(4-Bromophenyl)-N'-(4-fluorobenzoyl)-1*H*-pyrazole-5-carbohydrazide (3a)**

White powder, mp: 284-287^o^C. ^1^H NMR (300 MHz, DMSO): 7.29-7.33 (m, 2H), 7.39-7.42 (m, 1H), 7.52-7.55 (m, 1H), 7.57-7.59 (m, 1H), 7.70-7.71 (d, *J* = 3.0 Hz, 1H), 7.84 (s, 1H), 7.99 (s, 3H), 8.38 (s, 1H), 13.33 (s, 1H). MS (ESI): 403 (C_17_H_13_BrFN_4_O_2_, [M+H]^+^). Anal. Calcd for C_17_H_12_BrFN_4_O_2_: C, 50.64; H, 3.00; N, 13.90%. Found: C, 50.58; H, 2.89; N, 13.99%.

**3-(4-Bromophenyl)-N'-(4-chlorobenzoyl)-1*H*-pyrazole-5-carbohydrazide (3b)**

White powder, mp: 280-283^o^C. ^1^H NMR (300 MHz, DMSO): 7.33-7.35 (m, 3H), 7.40-7.43 (m, 2H), 7.57-7.58 (m, 2H), 7.80-7.81 (m, 2H), 8.09 (s, 1H), 8.29 (s, 1H), 13.31 (s, 1H). MS (ESI): 418 (C_17_H_13_BrClN_4_O_2_, [M+H]^+^). Anal. Calcd for C_17_H_12_BrClN_4_O_2_: C, 48.65; H, 2.88; N, 13.35%. Found: C, 48.77; H, 2.89; N, 13.52%.

**N'-(4-Bromobenzoyl)-3-(4-bromophenyl)-1*H*-pyrazole-5-carbohydrazide (3c)**

White powder, mp: 297-298^o^C. ^1^H NMR (300 MHz, DMSO): 7.05-7.06 (d, *J* = 3.0 Hz, 1H), 7.20-7.32 (m, 1H), 7.55-7.58 (m, 1H), 7.67-7.68 (m, 1H), 7.80-7.81 (m, 3H), 7.99 (s, 1H), 8.09-8.11 (m, 2H), 8.22 (s, 1H), 13.25 (s, 1H). MS (ESI): 462 (C_17_H_13_Br_2_N_4_O_2_, [M+H]^+^). Anal. Calcd for C_17_H_12_Br_2_N_4_O_2_: C, 43.99; H, 2.61; N, 12.07%. Found: C, 44.12; H, 2.78; N, 12.15%

**3-(4-Bromophenyl)-N'-(4-methylbenzoyl)-1*H*-pyrazole-5-carbohydrazide (3d)**

White powder, mp: 270-272^o^C. ^1^H NMR (300 MHz, DMSO): 3.62 (s, 3H), 7.01-7.04 (m, 2H), 7.48-7.51 (m, 2H), 7.52-7.54 (m, 2H), 7.77-7.79 (m, 1H), 7.91-7.96 (m, 3H), 8.33-8.36 (d, *J* = 9.0 Hz, 1H), 13.22 (s, 1H). MS (ESI): 399 (C_18_H_16_BrN_4_O_2_, [M+H]^+^). Anal. Calcd for C_18_H_15_BrN_4_O_2_: C, 54.15; H, 3.79; N, 14.03%. Found: C, 54.22; H, 3.90; N, 13.89%.

**3-(4-Bromophenyl)-N'-(4-methoxybenzoyl)-1*H*-pyrazole-5-carbohydrazide (3e)**

White powder, mp: 281-282^o^C. ^1^H NMR (300 MHz, DMSO): 3.82 (s, 3H), 7.00-7.04 (m, 2H), 7.25-7.28 (d, *J* = 9.0 Hz, 1H), 7.38-7.41 (m, 1H), 7.52-7.54 (m, 1H), 7.65-7.76 (m, 2H), 7.88-7.93 (m, 2H), 7.96-7.97 (d, *J* = 3.0 Hz, 1H), 8.24-8.26 (d, *J* = 6.0 Hz, 1H), 13.20 (s, 1H). MS (ESI): 415 (C_18_H_16_BrN_4_O_3_, [M+H]^+^). Anal. Calcd for C_18_H_15_BrN_4_O_3_: C, 52.06; H, 3.64; N, 13.49%. Found: C, 52.00; H, 3.51; N, 13.55%.

**3-(4-Bromophenyl)-N'-(4-nitrobenzoyl)-1*H*-pyrazole-5-carbohydrazide (3f)**

White powder, mp: 255-257^o^C. ^1^H NMR (300 MHz, DMSO): 7.05-7.06 (m, 2H), 8.30-8.33 (m, 2H), 8.37-8.38 (m, 1H), 8.69 (s, 1H), 8.70-7.73 (m, 3H), 8.89 (s, 1H), 8.90-8.91 (m, 1H), 13.05 (s, 1H). MS (ESI): 430 (C_17_H_13_BrN_5_O_4_, [M+H]^+^). Anal. Calcd for C_17_H_12_BrN_5_O_4_: C, 47.46; H, 2.81; N, 6.28%. Found: C, 47.55; H, 2.67; N, 6.33%.

**3-(4-Bromophenyl)-N'-(3-fluorobenzoyl)-1*H*-pyrazole-5-carbohydrazide (3g)**

White powder, mp: 278-280^o^C. ^1^H NMR (300 MHz, DMSO): 6.99-7.02 (m, 2H), 7.05-7.06 (d, *J* = 3.0 Hz, 1H), 7.38-7.43 (m, 3H), 7.55 (s, 1H), 7.67-7.68 (m, 1H), 7.80-7.82 (m, 2H), 8.09 (s, 1H), 13.35 (s, 1H). MS (ESI): 403 (C_17_H_13_BrFN_4_O_2_, [M+H]^+^). Anal. Calcd for C_17_H_12_BrFN_4_O_2_: C, 50.64; H, 3.00; N, 13.90%. Found: C, 50.72; H, 2.88; N, 14.03%.

**3-(4-Bromophenyl)-N'-(3-chlorobenzoyl)-1*H*-pyrazole-5-carbohydrazide (3h)**

White powder, mp: 246-248^o^C. ^1^H NMR (300 MHz, DMSO): 7.00 (s, 1H), 7.10-7.12 (m, 2H), 7.25-7.27 (d, *J* = 6.0 Hz, 1H), 7.31-7.33 (m, 1H), 7.47-7.48 (m, 2H), 7.80-7.81 (m, 2H), 7.99 (s, 1H), 8.07-8.08 (m, 1H), 13.22 (s, 1H). MS (ESI): 418 (C_17_H_13_BrClN_4_O_2_, [M+H]^+^). Anal. Calcd for C_17_H_12_BrClN_4_O_2_: C, 48.65; H, 2.88; N, 13.35%. Found: C, 48.54; H, 2.90; N, 13.19%.

**N'-(4-Bromobenzoyl)-3-(3-bromophenyl)-1*H*-pyrazole-5-carbohydrazide (3i)**

White powder, mp: 229-231^o^C. ^1^H NMR (300 MHz, DMSO): 7.13-7.15 (m, 3H), 7.35-7.36 (m, 2H), 7.60-7.61 (m, 1H), 7.67-7.68 (m, 2H), 7.80-7.82 (m, 1H), 8.20 (s, 1H), 8.22-8.23 (m, 1H), 13.33 (s, 1H). MS (ESI): 462 (C_17_H_13_Br_2_N_4_O_2_, [M+H]^+^). Anal. Calcd for C_17_H_12_Br_2_N_4_O_2_: C, 43.99; H, 2.61; N, 12.07%. Found: C, 43.87; H, 2.58; N, 12.15%

**3-(4-Bromophenyl)-N'-(3-methylbenzoyl)-1*H*-pyrazole-5-carbohydrazide (3j)**

White powder, mp: 287-288^o^C. ^1^H NMR (300 MHz, DMSO): 3.35 (s, 3H), 7.05-7.06 (d, *J* = 3.0 Hz, 1H), 7.30-7.33 (m, 2H), 7.69 (s, 1H), 7.87-7.88 (m, 1H), 7.93-7.95 (m, 2H), 7.99 (s, 1H), 8.12-8.14 (m, 2H), 8.22-8.23 (m, 1H), 13.20 (s, 1H). MS (ESI): 399 (C_18_H_16_BrN_4_O_2_, [M+H]^+^). Anal. Calcd for C_18_H_15_BrN_4_O_2_: C, 54.15; H, 3.79; N, 14.03%. Found: C, 54.02; H, 3.88; N, 14.11%.

**3-(4-Bromophenyl)-N'-(3-methoxybenzoyl)-1*H*-pyrazole-5-carbohydrazide (3k)**

White powder, mp: 251-253^o^C. ^1^H NMR (300 MHz, DMSO): 3.81 (s, 3H), 7.00-7.05 (m, 3H), 7.32-7.38 (m, 2H), 7.52-7.58 (m, 3H), 7.80-7.81 (m, 1H), 7.99 (s, 1H), 8.02 (s, 1H), 13.32 (s, 1H). MS (ESI): 415 (C_18_H_16_BrN_4_O_3_, [M+H]^+^). Anal. Calcd for C_18_H_15_BrN_4_O_3_: C, 52.06; H, 3.64; N, 13.49%. Found: C, 51.91; H, 3.79; N, 13.52%.

**3-(4-Bromophenyl)-N'-(3-nitrobenzoyl)-1*H*-pyrazole-5-carbohydrazide (3l)**

White powder, mp: 275-276^o^C. ^1^H NMR (300 MHz, DMSO): 7.33-7.35 (m, 1H), 8.15-8.16 (d, *J* = 3.0 Hz, 1H), 8.30-8.33 (m, 2H), 8.57-8.60 (m, 3H), 8.69 (s, 1H), 8.70-7.72 (m, 2H), 8.89 (s, 1H), 13.31 (s, 1H). MS (ESI): 430 (C_17_H_13_BrN_5_O_4_, [M+H]^+^). Anal. Calcd for C_17_H_12_BrN_5_O_4_: C, 47.46; H, 2.81; N, 6.28%. Found: C, 47.35; H, 2.97; N, 6.31%.

**3-(4-Bromophenyl)-N'-(2-fluorobenzoyl)-1*H*-pyrazole-5-carbohydrazide (3m)**

White powder, mp: 297-298^o^C. ^1^H NMR (300 MHz, DMSO): 6.83-6.85 (m, 1H), 7.00-7.03 (m, 2H), 7.15-7.16 (d, *J* = 3.0 Hz, 1H), 7.37-7.38 (m, 2H), 7.43-7.44 (m, 2H), 7.69 (s, 1H), 7.89-7.91 (m, 1H), 7.99-8.00 (m, 1H), 13.26 (s, 1H). MS (ESI): 403 (C_17_H_13_BrFN_4_O_2_, [M+H]^+^). Anal. Calcd for C_17_H_12_BrFN_4_O_2_: C, 50.64; H, 3.00; N, 13.90%. Found: C, 50.72; H, 2.91; N, 14.10%.

**3-(4-Bromophenyl)-N'-(2-chlorobenzoyl)-1*H*-pyrazole-5-carbohydrazide (3n)**

White powder, mp: 280-281^o^C. ^1^H NMR (300 MHz, DMSO): 7.00-7.01 (d, *J* = 3.0 Hz, 1H), 7.30-7.33 (m, 2H), 7.37-7.38 (m, 1H), 7.69 (s, 1H), 7.70-7.71 (m, 2H), 7.93-7.95 (m, 3H), 8.39 (s, 1H), 13.37 (s, 1H). MS (ESI): 418 (C_17_H_13_BrClN_4_O_2_, [M+H]^+^). Anal. Calcd for C_17_H_12_BrClN_4_O_2_: C, 48.65; H, 2.88; N, 13.35%. Found: C, 48.52; H, 3.00; N, 13.31%.

**N'-(4-Bromobenzoyl)-3-(2-bromophenyl)-1*H*-pyrazole-5-carbohydrazide (3o)**

White powder, mp: 281-283^o^C. ^1^H NMR (300 MHz, DMSO): 6.89 (s, 1H), 7.33-7.35 (m, 2H), 7.55-7.57 (d, *J* = 6.0 Hz, 1H), 7.60-7.63 (m, 2H), 7.75-7.77 (d, *J* = 6.0 Hz, 1H), 7.87-7.88 (m, 1H), 7.90-7.93 (m, 3H), 13.30 (s, 1H). MS (ESI): 462 (C_17_H_13_Br_2_N_4_O_2_, [M+H]^+^). Anal. Calcd for C_17_H_12_Br_2_N_4_O_2_: C, 43.99; H, 2.61; N, 12.07%. Found: C, 43.86; H, 2.78; N, 12.01%

**3-(4-Bromophenyl)-N'-(2-methylbenzoyl)-1*H*-pyrazole-5-carbohydrazide (3p)**

White powder, mp: 292-294^o^C. ^1^H NMR (300 MHz, DMSO): 3.66 (s, 3H), 7.00 (s, 1H), 7.10-7.13 (m, 2H), 7.22-7.24 (m, 2H), 7.35-7.36 (d, *J* = 4.0 Hz, 1H), 7.40-7.41 (m, 2H), 7.45-7.47 (d, *J* = 6.0 Hz, 1H), 7.69 (s, 1H), 7.99-8.00 (m, 1H), 13.18 (s, 1H). MS (ESI): 399 (C_18_H_16_BrN_4_O_2_, [M+H]^+^). Anal. Calcd for C_18_H_15_BrN_4_O_2_: C, 54.15; H, 3.79; N, 14.03%. Found: C, 54.08; H, 3.91; N, 14.12%.

**3-(4-Bromophenyl)-N'-(2-methoxybenzoyl)-1*H*-pyrazole-5-carbohydrazide (3q)**

White powder, mp: 287-290^o^C. ^1^H NMR (300 MHz, DMSO): 3.85 (s, 3H), 7.00-7.05 (m, 3H), 7.35-7.38 (m, 2H), 7.52-7.54 (d, *J* = 6.0 Hz, 1H), 7.65-7.66 (d, *J* = 3.0 Hz, 1H), 7.82-7.86 (m, 2H), 7.95-7.97 (m, 2H), 13.22 (s, 1H). MS (ESI): 415 (C_18_H_16_BrN_4_O_3_, [M+H]^+^). Anal. Calcd for C_18_H_15_BrN_4_O_3_: C, 52.06; H, 3.64; N, 13.49%. Found: C, 52.12; H, 3.69; N, 13.35%.

**3-(4-Bromophenyl)-N'-(2-nitrobenzoyl)-1*H*-pyrazole-5-carbohydrazide (3r)**

White powder, mp: 266-267^o^C. ^1^H NMR (300 MHz, DMSO): 7.15 (s, 1H), 7.55-7.56 (d, *J* = 3.0 Hz, 1H), 7.71-7.73 (d, *J* = 6.0 Hz, 1H), 7.80-7.83 (m, 2H), 7.97-7.98 (m, 1H), 8.20-8.21 (m, 1H), 8.25-8.26 (d, *J* = 3.0 Hz, 1H), 8.29-8.32 (m, 2H), 8.43-8.44 (m, 1H), 13.51 (s, 1H). MS (ESI): 430 (C_17_H_13_BrN_5_O_4_, [M+H]^+^). Anal. Calcd for C_17_H_12_BrN_5_O_4_: C, 47.46; H, 2.81; N, 6.28%. Found: C, 47.38; H, 2.87; N, 6.22%.

**N'-Benzoyl-3-(4-bromophenyl)-1*H*-pyrazole-5-carbohydrazide (3s)**

White powder, mp: 288-290^o^C. ^1^H NMR (300 MHz, DMSO): 6.99-7.00 (m, 1H), 7.01-7.03 (m, 2H), 7.05-7.06 (d, *J* = 3.0 Hz, 1H), 7.27-7.29 (m, 2H), 7.33 (s, 1H), 7.40-7.42 (m, 1H), 7.61-7.65 (m, 2H), 8.19 (s, 1H), 8.22-8.23 (m, 1H), 13.25 (s, 1H). MS (ESI): 385 (C_17_H_14_BrN_4_O_2_, [M+H]^+^). Anal. Calcd for C_17_H_13_BrN_4_O_2_: C, 53.00; H, 3.40; N, 14.54%. Found: C, 53.11; H, 3.29; N, 14.66%.

**The connection between theoretical results from docking calculations and X-ray data for compound 3n.**


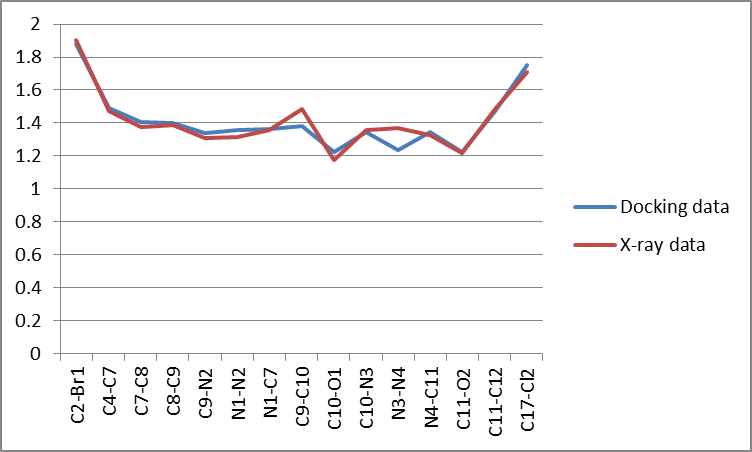


R = 0.9597

Graph 1.


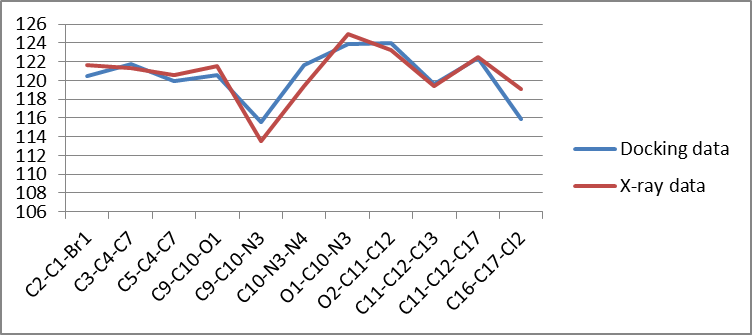


R = 0.8565

Graph 2.

**Correlations among antibacterial, anti DNA gyrase and CDOCKER-ENERGY**


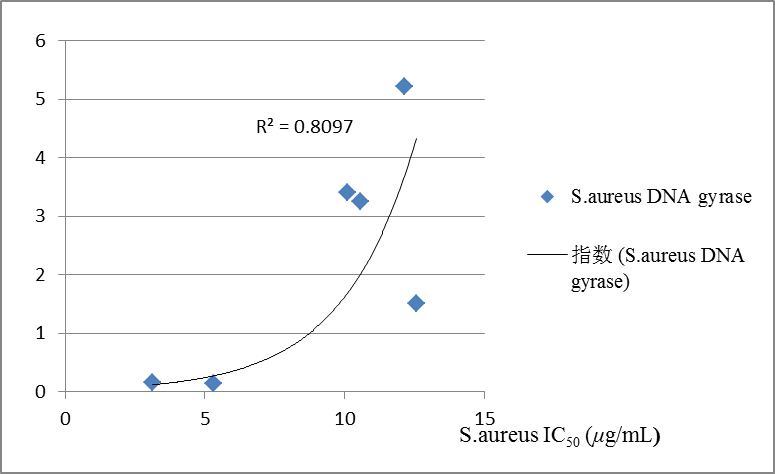


Graph 3.


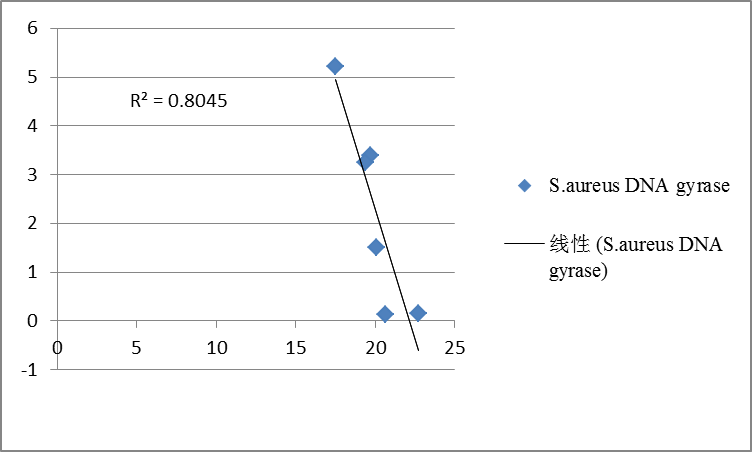


CDOCKER-ENERGY

Graph 4.
